# Domestic violence reporting during the COVID-19 pandemic: evidence from Latin America

**DOI:** 10.1007/s11150-022-09607-9

**Published:** 2022-04-30

**Authors:** Santiago M. Perez-Vincent, Enrique Carreras

**Affiliations:** 1 Innovation in Citizen Services Division (IFD/ICS), Inter-American Development Bank (IDB), 1300 New York Ave NW, Washington, DC 20577 USA; 2 Collegio Carlo Alberto—University of Turin, 8 Piazza Vincenzo Arbarello, 10122 Torino, Italy

**Keywords:** Domestic violence, Lockdown, COVID-19, Latin America, J12, J16, I18

## Abstract

This article examines changes in the frequency and characteristics of domestic violence reports following the onset of the COVID-19 pandemic and the imposition of mobility restrictions in six Latin American countries. We find significantly different patterns between reports of psychological and physical violence, non-cohabitant and cohabitant violence, and across alternative reporting channels (domestic violence hotlines, emergency lines, and police reports). Calls to domestic violence hotlines soared, suggesting that this channel was best suited to respond to victims’ needs during the pandemic. In turn, calls to emergency lines and police complaints declined (especially in the first weeks of the pandemic), consistent with an increase in the perceived (relative) cost of using these channels. The results reveal how the pandemic altered domestic violence victims’ demand for institutional help and highlight the relevance of domestic violence hotlines as an accessible and valuable service.

## Introduction

Crime and violence constitute a severe development challenge for Latin America and the Caribbean (LAC), a region where the per-capita homicide rate is four times higher than the global average (UNODC, 2019). Violence in domestic settings, including violence against partners, family members, and close individuals, is one of the most frequent manifestations of violence. According to recent estimates, in LAC, one in four women aged 15–49 years has experienced physical or sexual violence by an intimate partner in her lifetime (WHO, 2021). Domestic violence has several adverse effects on victims and their families: it impacts the health of newborns (Aizer, 2011); it alters victims’ economic decisions and opportunities (Borker, 2017; Siddique, 2018); and it increases the likelihood that children in the household will be abused or neglected, and that these children will reproduce violent behaviors in adulthood, extending the cycle of violence (Gage & Silvestre, 2010; Kimber et al., 2018). This wide range of effects carries high social and economic costs (Fearon & Hoeffler, 2014; Garcia-Moreno & Watts, 2011). The high prevalence of domestic violence and the magnitude of its negative consequences make its reduction a key development challenge for the region.

Designing policies to reduce domestic violence effectively requires a thorough understanding of its causes. The literature has identified several factors associated with the incidence of domestic violence, mainly distinguishing between fundamental and situational factors. Fundamental (or structural) factors refer to deeply rooted economic, social, or cultural drivers of violence, such as social norms about violence tolerance, economic gender inequality, and poverty (Gibbs et al., 2020; Jewkes, 2002). Situational factors refer to circumstances that can trigger violence, such as negative economic shocks (Aizer, 2010, 2011; Anderberg et al., 2016; Bhalotra et al., 2021; Bobonis et al., 2013; Buller et al., 2018; Munyo & Rossi, 2015; Pronyk et al., 2006); stress, anxiety, frustration, and depression (Brooks et al., 2020; Card & Dahl, 2011; Munyo & Rossi, 2013); exposure to perpetrators (Dugan et al., 1999; Peterman et al., 2020); substance abuse (Abramsky et al., 2011; Angelucci, 2008; Devries et al., 2014); pollution (Herrnstadt & Muehlegger, 2015); and other environmental factors (Henke & Hsu, 2020; Sanz-Barbero et al., 2018).

The mobility restrictions associated with the COVID-19 pandemic, together with the fear of contagion and voluntary social distancing, led to a drastic reduction in people’s mobility and disrupted global economic activity. These abrupt changes in social dynamics generated situational factors associated with domestic violence, leading to concern among authorities and civil society (U.N. Women 2020) and prompting several studies aimed at identifying the pandemic’s impact on domestic violence.

Measuring this impact is challenging for various reasons. First, domestic violence is a complex and heterogeneous issue, and the pandemic has evolved differently in diverse countries and settings. The relationship between these two complex phenomena (domestic violence and the pandemic) has nuances and subtleties. Second, measuring this impact requires reliable data, which is challenging given the high underreporting rates of domestic violence incidents. This issue is particularly relevant in the context of the pandemic, which may have altered the incentives and opportunities for victims to report incidents and the ability of response services to record them.

This complexity might explain the current ambiguous evidence on the evolution of domestic violence reporting during the pandemic: some studies showed no significant changes or found decreases (Ashby, 2020; Bullinger et al., 2021, for U.S. cities; Campedelli et al., 2020; Payne & Morgan, 2020, for Australia; Silverio-Murillo et al., 2020, for Mexico), while others found increases (Agüero, 2021, for Peru; Leslie and Wilson, 2020; Mohler et al., 2020, in the U.S.; Perez-Vincent et al., 2020, for Buenos Aires; Sanga & McCrary, 2020; Silverio-Murillo et al., 2020, for Mexico).

This study estimates the impact of the COVID-19 pandemic on the evolution of domestic violence reports using daily data for six Latin American countries: Argentina, Colombia, Costa Rica, Ecuador, Peru, and Uruguay. We use three types of data: calls to domestic violence hotlines (for the City of Buenos Aires in Argentina, Colombia, and Peru); calls to emergency lines (for Ecuador, Lima in Peru, and Costa Rica); and police/legal complaints (for Colombia, Ecuador, and Uruguay). To assess how the pandemic affected domestic violence reporting, we compare the daily number of reports before and after mid-March (i.e., the time of the year when the pandemic and mobility restrictions started) across multiple years.^1^ Formally, we use a linear regression model with week-of-the-year dummy variables to account for seasonal movements in the number of reports and year dummy variables to account for secular trends. Using this strategy, we obtain the pandemic’s impact on domestic violence reports under the assumption that, if the pandemic had not occurred, reports would have shown a seasonal evolution as in previous years. We complement this strategy with an event study model, which allows us to assess the validity of the previous assumption and observe how the pandemic’s impact evolved.

Using data through June 2020, we find that the pandemic’s impact on domestic violence reports varied significantly across countries, periods, types of violence, and reporting channels. We find an increase in calls to domestic violence hotlines (84 percent in Buenos Aires, Argentina; 127 percent in Colombia; and 16 percent in Peru) and a drop in calls to emergency lines (−16 percent in Ecuador; −10 percent in Costa Rica; and −53 percent in Lima, Peru) and police reports (−40 percent in Colombia, −41 percent in Ecuador, and −8 percent in Uruguay) from the imposition of mobility restrictions in March 2020 until June 2020.

The pandemic also affected the characteristics of domestic violence reporting. First, where information on the type of violence reported was available, we find that reports of psychological violence amplified (and led) the changes observed in total reports. Second, using data on the relationship between victim and perpetrator in Buenos Aires and Uruguay, we find that the change in domestic violence reports differed depending on whether victims and perpetrators lived together or not. Finally, relying on information from the City of Buenos Aires, we identify a change in the type of institutional response provided by the domestic violence hotline. Calls that resulted in providing information accounted for most of the overall increase in calls; however, police interventions following calls also went up significantly.

It is important to emphasize that the magnitude and mixed directions of these results are related to the impact that the pandemic had on the *reporting* (and *recording)* of domestic violence, not necessarily on the *incidence* of domestic violence events. Various factors could have affected reporting and not domestic violence itself (and vice versa). One possible explanation for the observed results is that the pandemic affected the relative incidence of different types of violence and the perceived costs and benefits of reporting them through alternative channels. These changes may have led to variations in the rate of reporting and the choice of reporting channels. Fear of contracting COVID-19 on entering a judicial process, mobility restrictions, or increased household economic insecurity may have reduced the likelihood that a person would decide to report a domestic violence incident to law enforcement authorities. The pandemic’s impact on the decision to report or not (and the choice of the channel) may also have depended on the perceived severity or type of incident. The relatively greater shifts observed in reports of psychological violence align with victims considering these types of incidents less urgent and preferring to avoid initiating legal processes in turbulent and uncertain times such as those brought on by the pandemic. Increases in calls to domestic violence hotlines suggest that this channel was best suited to respond to the demand from domestic violence victims during the pandemic. In turn, the drop in calls to comprehensive emergency lines and in legal complaints is consistent with an increase in the perceived (relative) cost of using these channels during this period.

This study adds to the understanding of the relationship between the COVID-19 pandemic and domestic violence in various ways and delivers important policy implications. First, it provides evidence for several Latin American countries, broadening the geographic scope of this literature, which has focused primarily on the United States. Second, drawing on a rich database of domestic violence reports for several countries and reporting channels, it provides new insights into how the pandemic affected domestic violence reporting. We found that the impact of the pandemic on domestic violence reporting varied significantly depending on the type of violence reported, the relationship between victim and perpetrator, and the reporting channel used. These heterogeneous effects may help make sense of some of the ambiguous results found in the literature. The wide range of results also cautions against overstating the external validity of existing studies. It highlights the importance of examining further contexts and data sources to better understand domestic violence’s evolution during the pandemic (and obtain better insights into the drivers of domestic violence and its reporting). Finally, the findings highlight the relevance of domestic violence hotlines as an accessible and valuable service for providing institutional help to victims. During the first months of the pandemic, hotlines managed to respond to victim demand for institutional help and bring this help closer to them. This dynamic emphasizes the importance of having and strengthening domestic violence hotlines.

The remainder of this paper is structured as follows: Section II summarizes the available evidence on the impact of the pandemic on domestic violence and highlights the challenges faced by such studies. Section III describes the databases used. Section IV comments briefly on the mobility restrictions imposed in each of the countries included in the analysis. Section V explains the empirical method used to estimate the impact of the pandemic on domestic violence reporting. Section VI describes the impact on reporting frequency, and Section VII describes the changes in reporting characteristics. The final section presents the main conclusions of the study.

## COVID-19 pandemic and domestic violence: available evidence and empirical challenges

The COVID-19 pandemic altered almost every dimension of people’s lives. As the virus spread, governments imposed mandatory mobility restrictions to reduce contagion. These policies were implemented—with different stringency levels—in almost every country. Together with the fear of contagion and voluntary social distancing, the imposition of such restrictions led to a drastic reduction in people’s mobility and disrupted global economic activity. These disruptions generated conditions associated with domestic violence.

Lockdowns can lead to diverse psychological effects, including stress, anxiety, trauma, irritation, and depression (Brooks et al., 2020). Recent studies predicted severe mental health consequences from this pandemic (Galea et al., 2020; Pfefferbaum & North, 2020). Stress, frustration, and emotional instability are situational factors related to violence (Munyo & Rossi, 2013) and particularly to domestic violence (Card & Dahl, 2011). The economic effects of the pandemic could also impact the incidence of domestic violence. The COVID-19 pandemic increased unemployment and economic distress (Bitler et al., 2020) and gender inequality (Alon et al., 2020; Dang & Nguyen, 2021). In Latin America, women’s participation in the labor force shrunk starkly during the pandemic (ECLAC, 2021). Changes in the unemployment rate (Anderberg et al., 2016), the gender income gap (Aizer, 2010), and access to finance or employment opportunities (Pronyk et al., 2006) might affect economic security, alter the intrahousehold balance, and lead to increases in domestic violence (Buller et al., 2018). Moreover, unemployment and lockdowns increase the time people spend at home, potentially increasing victims’ exposure to perpetrators (Dugan et al., 1999).

The presence of these situational triggers motivated several studies that sought to assess the impact of the pandemic on domestic violence. This task presents three main challenges that make it difficult to draw accurate conclusions. These challenges highlight the need to use different approaches and sources of information and examine various scenarios.

First, domestic violence is a complex and heterogeneous issue, and the pandemic has evolved differently in diverse countries and settings. The relationship between these two complex phenomena (domestic violence and the pandemic) has nuances and subtleties. Looking at the evolution of domestic violence reports for different periods, types of violence, or reporting channels may result in different (and even opposite) conclusions, as we discuss in subsequent sections.

Second, measuring this impact requires reliable data, which is challenging given the high underreporting rates of domestic violence incidents. Misreporting is a widely recognized problem in domestic violence quantitative studies (Cullen, 2020; Palermo et al., 2015). This issue is particularly relevant in the context of the pandemic, which may have altered the incentives and opportunities for victims to report incidents. On the one hand, the pandemic (and the subsequent mobility restrictions) might have increased the perceived costs of reporting domestic violence incidents. Lockdowns restricted leaving the house and increased exposure to perpetrators. Cohabiting with the perpetrator may have impeded looking for help or made it too risky for the victims. Furthermore, victims may not have wanted to contact law enforcement and go through the legal process for fear of COVID-19. The increased economic insecurity associated with the pandemic may also have deterred victims from reporting incidents to avoid potential loss of income due to the eventual arrest or incapacitation of the perpetrator. This fear may be more significant under strict penalty plans. Victims’ willingness to report arguably decreases as victims lose control of the procedures carried out after reporting an incident (Goodmark, 2018). By nature, this potential increase in underreporting is worrying as it makes measuring domestic violence more difficult and it increases the risk of domestic violence (Miller & Segal, 2019). On the other hand, advertising campaigns warning about the risk of domestic violence incidents during the pandemic and a heightened presence of the topic in the media may have prompted victims to report more incidents. Also, by increasing the time spent at home, mobility restrictions might have increased neighbors’ or family members’ awareness of domestic violence incidents that had previously gone unnoticed. Increased surveillance by neighbors may lead to more domestic violence reports (Perez-Vincent et al., 2020). In short, the pandemic likely altered both the frequency of domestic violence incidents and the likelihood of them being reported, making assessing the pandemic’s impact on domestic violence challenging.

Third, the pandemic might have altered response services’ operations since emergency and victim response services had to adjust their practices to prevent or respond to COVID-19 among personnel. These adjustments may have affected (at least momentarily) their ability to support and respond to victims and any reduced response capacity might have decreased the number of domestic violence incidents recorded. We find evidence of these changes in the evolution of the *effective response rate* (i.e., the share of answered calls relative to total received calls) on Costa Rica’s emergency line. Costa Rica experienced a decline in the first months of the pandemic, with the rate falling from 91 percent in January 2020 to a low of 46 percent in July 2020. Two main factors seem to have driven this decline. On the one hand, the increase in COVID-19-related calls, such as medical emergencies and complaints about citizens violating mobility restrictions, reduced the capacity to answer other calls. On the other hand, some operators caught COVID-19 and had to be isolated, resulting in an operator shortage.^2^

In addition to these forced changes in operations, the anticipation of possible increases in domestic violence incidents may have led governments to strengthen the response capacity of some of these services. Increased capacity might have translated into a mechanical increase in recorded events, if previously there was not enough capacity to register every incident.

In summary, the pandemic might have affected the capacity of response services to record incidents, adding another confounding factor in the quantitative analysis of the pandemic’s impact on domestic violence.

The ambiguity of the existing evidence on the pandemic’s impact on the incidence of domestic violence might in part be due to these three factors (i.e., the complexity of domestic violence, the reliability of available data, and adjustments to services due to the pandemic). In each study, the observed result is a combination of the impact on different types of domestic violence, each with its own dynamics, and the potential impact of changes in reporting and recording rates.^3^ With these caveats in mind, we nevertheless identify some trends among groups of studies that focused on similar sources of information.

The first group of studies used data from official reports recorded by law enforcement agencies. They generally observed no significant changes or found decreases in the number of recorded domestic violence incidents (Ashby, 2020; Bullinger et al., 2021, for U.S. cities; Campedelli et al., 2020; Payne & Morgan, 2020, for Australia; Silverio-Murillo et al., 2020, for Mexico). The second group of studies used data from emergency lines (Leslie & Wilson, 2020; Mohler et al., 2020; Richards et al., 2021, in the U.S.; Sanga & McCrary, 2020) and specific domestic violence hotlines (Agüero, 2021, for Peru; Perez-Vincent et al., 2020, for Buenos Aires; Silverio-Murillo et al., 2020, for Mexico; Richards et al., 2021, for the U.S.). For the most part, these studies found increases in the number of calls.

Our study complements both sets of studies, providing novel evidence on the evolution of domestic violence reports during the COVID-19 pandemic for six Latin American countries using different data sources. We document how the pandemic changed the reporting of domestic violence incidents in countries for which there was limited to no previous evidence (Colombia, Costa Rica, Ecuador, and Uruguay) and extend and refine findings in previous papers that examine the cases of Argentina (Perez-Vincent et al., 2020) and Peru (Agüero, 2021).^4^

## Data: domestic violence reports

We collected administrative records of domestic violence incidents in six Latin American countries. We gathered three types of data: effective calls^5^ to domestic violence hotlines (Buenos Aires in Argentina, Colombia, and Peru), effective calls to emergency lines (Ecuador, Lima in Peru, and Costa Rica), and police/legal complaints (Colombia, Ecuador, and Uruguay).^6^

The datasets differ in the information they provide on reported incidents and the time frame they cover. Still, in all cases, the available information allows us to calculate the daily count of recorded domestic violence incidents from, at least, the beginning of 2019. Table 1 summarizes the scope of the different datasets. Unfortunately, there is no overlap across countries in multiple data sources.

**Table 1:** Summary of data sources

Data Source	Argentina	Colombia	Costa Rica	Ecuador	Uruguay	Peru
Police Reports	–	2018–2020	–	2018–2020	2019–2020	–
Emergency Line	–	–	2019–2020	2018–2020	–	2019–2020
Domestic Violence Hotline	2017–2020	2018–2020	–	–	–	2018–2020

*Source:* Colombia: Policía Nacional (police reports) and Línea 155 (domestic violence hotline); Uruguay: Ministerio del Interior (police reports); Ecuador: Fiscalía General (police reports) and ECU911 (emergency line); Costa Rica: Ministerio de Seguridad and Instituto Nacional de Mujeres (emergency line); Lima, Peru: Línea 105 (emergency line) and Línea 100 (domestic violence hotline); Buenos Aires, Argentina: Línea 137 (domestic violence hotline)

For Argentina, we obtained administrative records on calls to Línea 137, a service line for victims and witnesses of domestic violence, in the City of Buenos Aires from the beginning of 2017 to June 30, 2020.^7^ This dataset is probably the most detailed. For each call, it provides demographic characteristics of the caller, how they relate to the victim, the type of violence being reported, the relationship between the victim and the offender, and demographic information for the victim and perpetrator. The database also includes details on the actions taken in response to the call, including requesting a police intervention, referring to other government services, or providing information.

For Colombia, we obtained information from two different sources. The Colombian National Police provided administrative records of domestic violence incidents reported to the police from January 1, 2019, until June 23, 2020, for Pereira, Barranquilla, Cali, Bogotá, Cartagena, Cúcuta, Medellin, and Pasto. These records also include information on the geographic location of the event within those cities. We complemented these data with information on calls to Línea 155, a hotline for women victims of domestic violence. This dataset contains the number of daily calls received by the hotline from January 1, 2018, to June 30, 2020, but provides no details about the calls.

For Costa Rica, we use administrative records on calls reporting domestic violence incidents received by the national emergency line (911) from January 1, 2019, to June 30, 2020. These records, provided by the Ministry of Security and the National Women’s Institute (INAMU), contain information on the type of violence reported (distinguishing between physical, psychological, and sexual forms of violence).

For Ecuador, we gathered two different datasets. First, we collected information on calls reporting domestic violence incidents received by the national emergency line (ECU911). We complemented this with data on official domestic violence reports (police/legal complaints) collected by the General Attorney’s Office (*Fiscalía General*), which contains information on the type of violence reported (also distinguishing between physical, psychological, and sexual forms of violence). Both datasets provide information for January 1, 2018, to June 30, 2020.

For Uruguay, we collected information on official domestic violence reports received by the Ministry of the Interior (which we refer to as “police” or “legal” complaints). This dataset contains detailed information on the relationship between victim and perpetrator for each reported incident.

Lastly, for Peru, we collected information from the Ministry of the Interior regarding calls reporting domestic violence incidents received by the police emergency line (Línea 105) from January 1, 2018, to June 30, 2020. While the hotline has national coverage, calls refer mainly to Lima. We complement this dataset with publicly available information on calls received by Línea 100, the domestic violence hotline in the whole country.^8^ The database provides demographic characteristics of the caller, how they relate to the victim, the type of violence reported, the relationship between the victim and the offender, and demographic information of victim and perpetrator.

The datasets contain information on the demand for different emergency, law enforcement, and domestic violence victim support services. The amount and dynamics of the reports received by these different agencies vary significantly. Table 2 summarizes the average daily reports recorded in the different databases by year, and before and after mobility restrictions took place in 2020. The numbers show some distinct patterns across different types of data sources. Figure 5 in the appendix shows the change in the number of reports registered in 2020 after the mobility restrictions (until June 30) for each of the data sources. Figure 6 in the appendix plots the weekly averages of the number of daily reports for each source.

**Table 2:** Summary statistics: average daily calls

			2017	2018	2019	2020		
						Pre-Lockdown	Lockdown	% Change
Domestic Violence Hotlines	Buenos Aires (Argentina)	Mean	27.1	22.1	21.4	16.6	26.9	62.0
		SD	0.45	0.39	0.34	0.58	0.71	
		Obs	365	365	365	79	103	
	Colombia	Mean		67.8	54.4	59.6	118	98.0
		SD		0.98	0.69	1.76	2.62	
		Obs		365	365	84	98	
	Peru	Mean		212.9	328.2	467.4	648.5	38.7
		SD		4.45	4.92	18.2	24	
		OBS		357	365	75	107	
Emergency Lines	Ecuador	Mean		294.0	344.3	347.1	285.3	−17.8
		SD		6.12	6.58	16.49	9.58	
		Obs		365	365	71	111	
	Costa Rica	Mean		127.7	108.1	116.7	108.3	−7.2
		SD		1.78	1.64	3.49	2.43	
		Obs		365	365	82	100	
	Lima (Peru)	Mean		166.2	162.2	209.9	92.6	−55.9
		SD		5.76	2.51	6.53	3.54	
		Obs		61	365	75	107	
Police Reports	Colombia	Mean		130.6	146.1	162.1	104.4	−35.6
		SD		1.62	1.96	5.48	3.50	
		Obs		365	365	84	91	
	Ecuador	Mean		111.2	88.0	85.6	61.3	−28.4
		SD		1.44	1.11	2.35	3.45	
		Obs		365	365	71	111	
	Uruguay	Mean		174.3	173.1	195.8	156.4	−20.1
		SD		1.53	1.65	3.87	2.04	
		Obs		365	365	72	110	

*Source:* Authors’ calculations based on data from the following sources: Colombia: *Policía Nacional* (police reports) and Línea 155 (domestic violence hotline); Uruguay: *Ministerio del Interior* (police reports); Ecuador: *Fiscalía General* (police reports) and ECU911 (emergency line); Costa Rica: *Ministerio de Seguridad* and *Instituto Nacional de Mujeres* (emergency line); Lima, Peru: Línea 105 (emergency line) and Línea 100 (domestic violence hotline); Buenos Aires, Argentina: Línea 137 (domestic violence hotline)

*Note:* Lockdown period until June 30, 2020. “% Change” refers to the percentage change in the average number of reports between the 2020 pre-lockdown period and the 2020 lockdown period (until June 30, 2020)

The average number of calls to the domestic violence hotlines (i.e., Línea 137 in Buenos Aires, Línea 155 in Colombia, and Línea 100 in Peru) rose after the mobility restrictions came into effect. Calls to national emergency lines tell a different story. Domestic violence calls to ECU911 in Ecuador and Línea 105 in Lima, Peru, fell after the mobility restrictions came into effect, and domestic violence calls to the emergency line (911) in Costa Rica remained relatively stable after the onset of the pandemic. The dynamics of police/judicial complaints are similar to that of emergency lines. Official domestic violence complaints in Colombia, Ecuador, and Uruguay fell after the lockdown came into place.

This first examination of the data suggests an increase in the use of the domestic violence hotlines and a decrease in the use of other reporting mechanisms during the first months of the pandemic. However, a more rigorous analysis is necessary to distinguish these changes from seasonal variations and to more accurately estimate the pandemic’s impact on the domestic violence reports. We discuss the methodology in Section V.

## Mobility restrictions

**Fig. 1:**
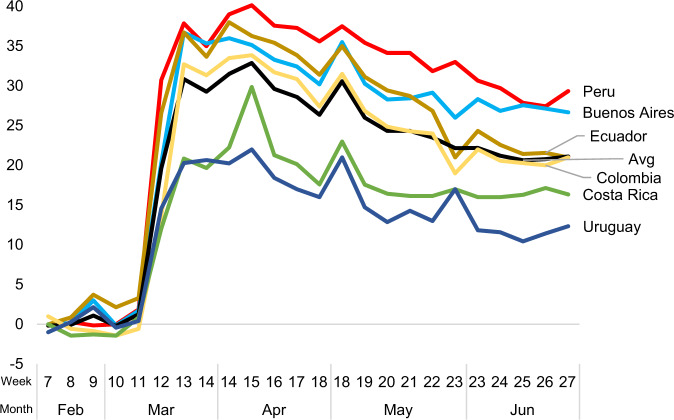
Percent Change in Time Spent at Home, Weekly Averages. *Source:* Google Mobility Report. The graph shows the percentage change in mobility in residential areas with respect to the average registered that same day of the week during the period January 3 to February 6, 2020

**Fig. 2:**
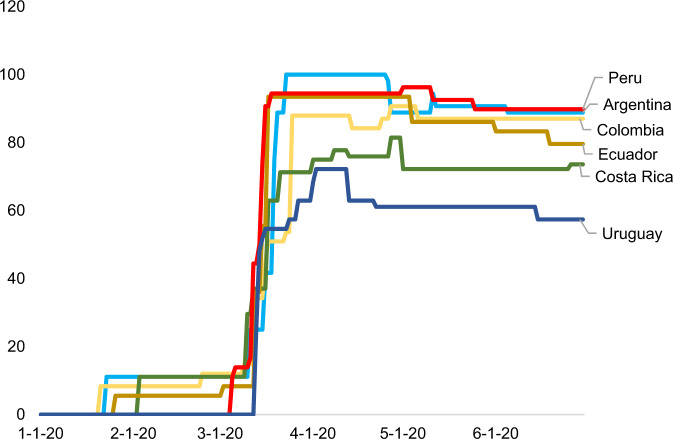
Evolution of the OxCGRT Stringency Index. *Source:* Oxford COVID-19 Government Response Tracker. The index considers the imposition of school closings, workplace closings, cancellation of public events, restrictions on gatherings, cancellation of public transport, stay-at-home requirements, restrictions on internal movement, international travel control, and public health campaigns

Countries across Latin America implemented different strategies to combat the effects of the pandemic, with varying degrees of government involvement and stringency in the policies they imposed. In all cases, however, some form of mobility restriction was implemented in mid-late March 2020. Figure 1 shows the evolution of the change in time people spent at home from February to June 2020 compared to normal levels, according to the Google Mobility Report.^9^ The relative changes in the time people spent at home in the different countries are consistent with the evolution of the stringency levels of the restrictions imposed by each government, measured by the Oxford COVID-19 Government Response Tracker (OxCGRT) Stringency Index.^10^ The OxCGRT Stringency Index provides a measure of the strictness of the prevailing mobility restrictions based on information on school closings, workplace closings, cancellation of public events, restrictions on gatherings, cancellation of public transport, stay-at-home requirements, restrictions on internal movement, international travel control, and public health campaigns (Hale et al. 2020) (Fig. 2).

Among the countries included in our study, Peru had the largest reduction in mobility. The Government of Peru established a stringent national quarantine on March 16, 2020, with only a few specific exceptions for essential workers.

The reduction in mobility was also relatively large in Buenos Aires, Argentina. The Government of Argentina decreed a national quarantine on Friday, March 20, 2020. The lockdown came into effect at a relatively early stage of the spread of the virus in the country.

In Ecuador, the government established a national quarantine on Sunday, March 12, 2020. The quarantine included the closure of schools and the suspension of massive events. On Tuesday, March 17, 2020, the mobility restrictions intensified as a partial curfew was implemented. The reduction in mobility during the first months of the pandemic was consistently higher than the average for the countries in our study.

In Colombia, the government established a stringent national quarantine on Wednesday, March 25, 2020, that only allowed people to leave the house for vital reasons. The measure was adopted relatively late (compared to the other countries), and the pandemic had already affected citizens’ mobility. Colombia’s mobility evolved in a similar way to the average of the studied countries.

The Government of Costa Rica established a national emergency on Monday, March 16, 2020, requiring schools to close and limiting immigration. The country’s mobility was one of the least affected in the region because the government used a less stringent approach to fight the pandemic. Costa Rica experienced a late surge in the disease in June.^11^

In Uruguay, national authorities started introducing mobility restrictions to combat the pandemic on March 16, 2020. Travel restrictions were imposed, and the population was advised to stay at home. Among the set of countries included in our analysis, Uruguay had the least stringent approach and, at least during the first months of the pandemic, showed the smallest change in people’s mobility.

## Empirical strategy

We estimate the pandemic’s impact on the frequency of different types of domestic violence reports using a linear regression model that includes time dummies to control for seasonal variations and secular trends in the dependent variable. Formally, we estimate the following model:

1 $$DVReports_d = \alpha + \beta \ast Pandemic + \partial _{dow} + \emptyset _{woy} + \gamma _{year} + \varepsilon _d$$

We use as dependent variables (*DV Reports*_*d*_) the number of calls for each day and the inverse hyperbolic sine (IHS). The IHS transformation (Johnson, 1949) is helpful since it approximates the logarithmic function but, contrary to it, is defined at zero (see Burbidge et al. 1988). The variable “Pandemic” is a dummy indicator that equals one for all days coming after the imposition of COVID-19 pandemic-related mobility restrictions.^12^ Our coefficient of interest is *β*, which represents the change in reports during the pandemic. When using the IHS transformation, the *β* coefficients can be easily re-expressed to percentage changes.^13^ Coefficients *∂*_*dow*_ are day-of-the-week fixed effects that capture systematic within-week patterns in the number of calls. We also include year fixed effects (*γ*_*year*_) to capture secular trends and week-of-the-year fixed effects (*∅*_*woy*_) to capture seasonal movements.

This model allows us to estimate the pandemic’s impact on the frequency of domestic violence reports under the parallel trends assumption that, if the pandemic had not occurred, the number of calls would have shown a seasonal evolution as in the previous years. This approach, used in previous work on domestic violence and the pandemic (Agüero, 2021; Leslie & Wilson, 2020), relies on comparing the change in the number of reports before and after the time of the imposition of mobility restrictions (mid-March) across multiple years. We estimate this model using information from January 1 to June 30 for the available years in each data set.

We present the ordinary least squares (OLS) estimates of the model using heteroscedasticity robust standard errors. We note that the time-series nature of our data could lead to autocorrelation in the model residuals and induce bias in the estimation of our standard errors. We use a heteroscedasticity and autocorrelation consistent (HAC) estimator for the standard errors to address this issue, following Newey-West (1987). This estimator allows us to perform (asymptotically) valid statistical inference in the presence of autocorrelation and heteroscedasticity.^14^ Additionally, since we are dealing with count data, we estimate negative binomial and Poisson specifications (Greene, 2008). As the results using Newey-West HAC standard errors and non-linear specifications are extremely similar to the standard linear model estimated by OLS, we present the results of this model throughout the document.

To check the validity of our design (i.e., examine the plausibility of the parallel trends assumption) and assess how the pandemic’s impact evolved, we also estimate an event study model using the following equation:

2 $$\begin{array}{l}DVReports_d =\alpha + \mathop {\sum}\nolimits_{p = 1}^3 {\left( {\beta _p \ast Period_p} \right)}\\ \qquad\qquad\qquad\quad\,+\,\mathop {\sum}\nolimits_{p = 5}^{13} {\left( {\beta _p \ast Period_p} \right)} + \partial _{dow} + \emptyset _{woy} + \gamma _{year} + \varepsilon _d\end{array}$$

Where *Period*_*p*_ refers to each two-week period from January 1 to June 30 in 2020, and the *β*_*p*_ coefficients trace out systematic changes in the number of domestic violence calls through the two-week periods in 2020 relative to the same period in previous years. The fourth two-week period (i.e., the second half of February) is the omitted category. We include the same set of fixed effects from Eq. (1).

The event study enables us to identify if, in the months before the pandemic (i.e., January to mid-March), the temporal dynamics of domestic violence reports in 2020 were similar to the ones in previous years. To do so, we assess if the coefficients for those two-week periods show any systematic pattern. Finding consistently significant coefficients for those (pre-pandemic) periods would indicate that the seasonal evolution of domestic violence reporting in 2020 was being different from that of previous years and would challenge the validity of the parallel trends assumption.

The following section shows the OLS estimates of these models using information for the number of domestic violence reports for each country and reporting channel.

## The impact of the pandemic on the frequency of domestic violence reports

In this section, we assess the impact of the pandemic on the number of calls to domestic violence hotlines in the City of Buenos Aires (Argentina), Colombia, and Peru; on domestic violence calls to emergency lines in Ecuador, Lima (Peru), and Costa Rica; and on domestic violence police complaints in Colombia, Ecuador, and Uruguay.

### Event study model: assessing the validity of the empirical design

**Fig. 3:**
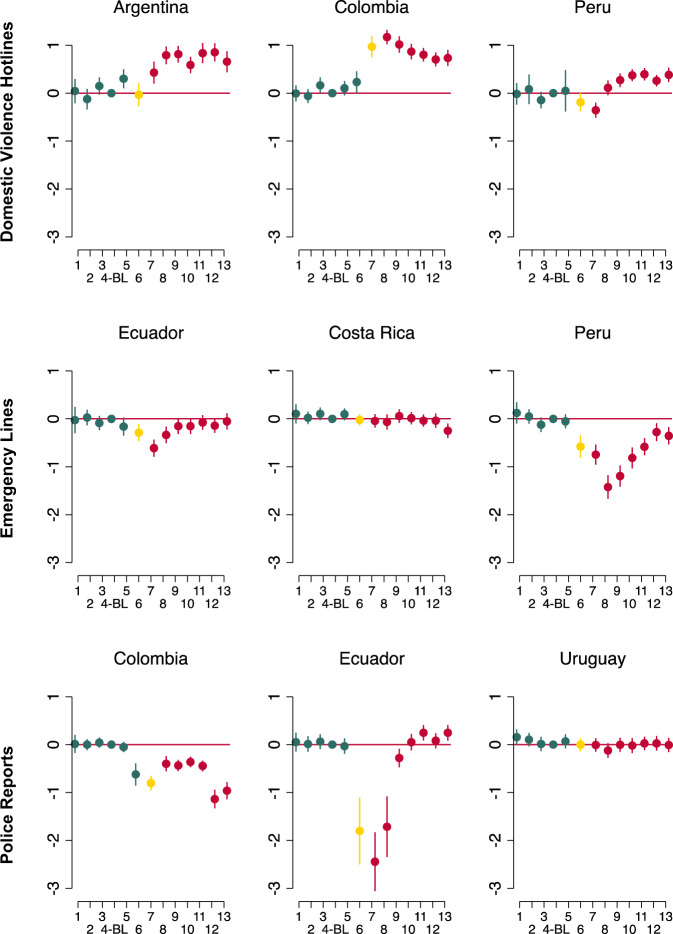
Impact of the Pandemic on Domestic Violence Reports: Event Study Model. *Source:* Authors’ calculations based on data from sources described in Section III. *Note:* The charts report the OLS estimates of the event study model (Eq. (2)) coefficients and their 95-percent confidence interval using the IHS transformation on the number of reports as dependent variable. Each coefficient represents a two-week period. The fourth two-week period (i.e., the second half of February) is the omitted category, our baseline (B.L.)

We assess the validity of the empirical approach using the event study model described in the previous section (Eq. 2). Figure 3 summarizes the estimation results using the IHS transformation of the number of calls as the dependent variable. The figure also reveals the dynamics of the impact of the pandemic on domestic violence reports.

Regarding calls to domestic violence hotlines, the event study model shows that, before the onset of the pandemic and the mobility restrictions, the trend for calls was similar to previous years (with coefficients not systematically different from zero). Following the onset of the pandemic, we observe a large, positive, and growing impact on the number of calls in Argentina and Peru and a positive and sustained impact in Colombia.

In terms of calls to emergency lines, the event study model allows us to verify that, before the pandemic, calls showed a trend parallel to that of previous years. In general, the coefficients for the first fortnights of the year are small in absolute value and are not statistically significant. After the onset of the pandemic, we find a large and sharp drop in calls in Lima, Peru, and Ecuador that attenuated over time. The dynamics in Costa Rica were different. In Costa Rica, the drop in the number of calls was generally smaller but became more pronounced over time (especially toward the end of the analysis period). There may be different explanations for this result. On the one hand, Costa Rica was one of the least affected countries during the first months of the pandemic, experiencing a relatively late outbreak of the disease, which may have also delayed the impact on domestic violence reports. On the other hand, as mentioned in Section II, the operational capacity of the hotline declined during the pandemic, especially in June and July. Response rate can impact the number of calls received, reflecting one of the possible biases that exist when trying to capture the evolution of domestic violence through call records.

The dynamics of the number of police or legal complaints were similar to that of calls to emergency lines. The event study shows that, for the most part, estimates before the imposition of mobility restrictions are not statistically significant. The only exception is Colombia, where there was a drop in the number of reports in the fortnight before the start of mobility restrictions. This early drop may be related to the decline in mobility that occurred even before mobility restrictions were imposed (as discussed in Section IV). In Colombia, the drop in the number of police complaints became more pronounced in June. In Uruguay, where mobility fell the least among the countries included in our study, judicial domestic violence complaints showed relatively small declines, which became smaller and not statistically significant as the pandemic progressed. In Ecuador, a sharp decline in the number of judicial domestic violence complaints was observed in the first month after the imposition of mobility restrictions. This large drop quickly attenuated and, by the end of June, the number of complaints increased (which might be partly explained by the late reporting of incidents occurring in the previous two months). This change in the direction of the impact is not unprecedented in LAC. In Mexico, Silverio-Murillo et al. (2020) found a short-term decline, followed by a spike in police reports.

Overall, the results of the estimation of the event study model show that, in the months before the pandemic (i.e., January to mid-March), the temporal dynamics of domestic violence reports in 2020 was similar to the one in previous years, providing support to the validity of the parallel trends assumption.

### Main results: how the pandemic changed the frequency of different domestic violence reports in LAC

**Fig. 4:**
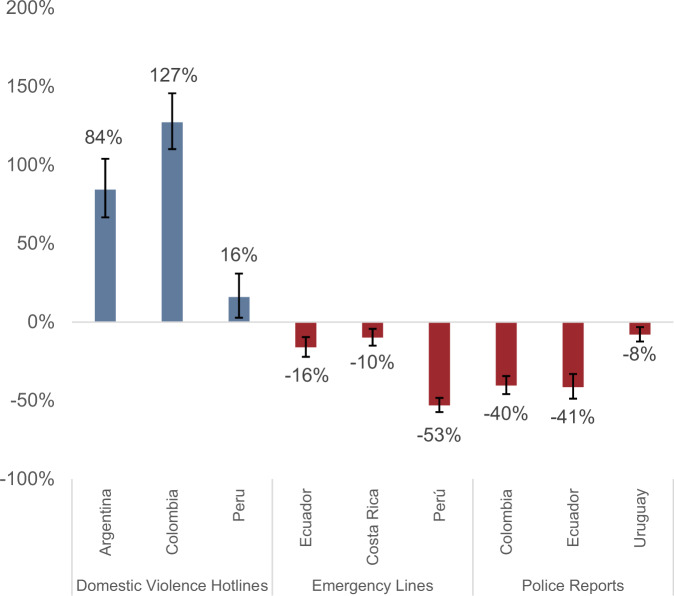
Impact of the Pandemic on Domestic Violence Reports. *Source:* Authors’ calculations based on data from the following sources: Colombia: Policía Nacional (police reports) and Línea 155 (domestic violence hotline); Uruguay: Ministerio del Interior (police reports); Ecuador: Fiscalía General (police reports) and ECU911 (emergency line); Costa Rica: Ministerio de Seguridad and Instituto Nacional de Mujeres (emergency line); Lima, Peru: Línea 105 (emergency line) and Línea 100 (domestic violence hotline); Buenos Aires, Argentina: Línea 137 (domestic violence hotline). *Note:* The figure presents the results of the OLS estimation of the β coefficient in Eq. (1) for each of the datasets examined, using the IHS transformation of the daily number of reports as the dependent variable. We re-express the *β* coefficient as a percentage change following Bellemare and Wichman (2020). 95% confidence intervals reported for each estimate

**Fig. 5:**
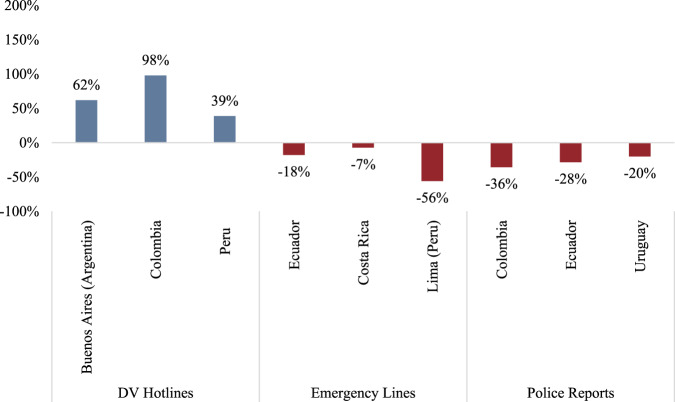
Domestic Violence Reports in 2020 Before and After the Pandemic (% Change). *Source:* Authors’ calculations based on data from the following sources: Colombia: *Policía Nacional* (police reports) and Línea 155 (DV hotline); Uruguay: *Ministerio del Interior* (police reports); Ecuador: *Fiscalía General* (police reports) and ECU911 (emergency line); Costa Rica: *Ministerio de Seguridad* and *Instituto Nacional de Mujeres* (emergency line); Lima, Peru: Línea 105 (emergency line) and Línea 100 (DV hotline); Buenos Aires, Argentina: Línea 137 (DV hotline). *Note:* Lockdown period until June 30, 2020. Figures show the percentage change in the average number of reports between the 2020 pre-lockdown period and the 2020 lockdown period (until June 30, 2020)

**Fig. 6:**
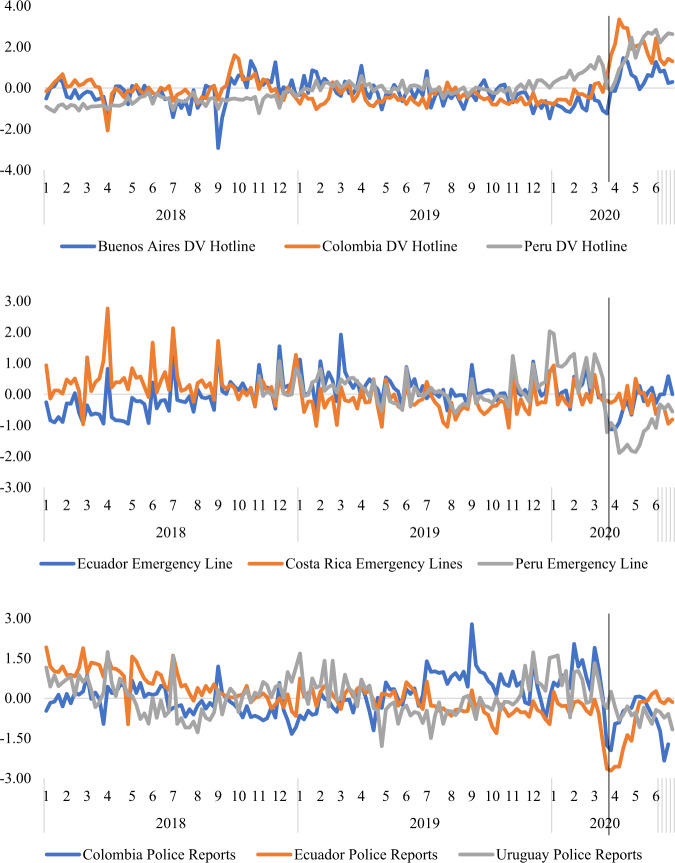
Domestic Violence Reports (Weekly Averages). *Source:* Colombia: Policía Nacional (police reports) and Línea 155 (DV hotline); Uruguay: Ministerio del Interior (police reports); Ecuador: Fiscalía General (police reports) and ECU911 (emergency line); Costa Rica: Ministerio de Seguridad and Instituto Nacional de Mujeres (emergency line); Lima, Peru: Línea 105 (emergency line) and Línea 100 (DV hotline); Buenos Aires, Argentina: Línea 137 (DV hotline)

Figure 4 and Table 3 summarize the overall impacts of the pandemic on the number of domestic violence reports. We group the results by type, distinguishing between domestic violence-specific hotlines, emergency lines, and police (or legal) complaints.

**Table 3:** Impact of the pandemic on domestic violence reports

Type	City or Country	Estimated Effect (*β*)			
		Calls		IHS (Calls)	
		Coef.	S.E.	Coef.	S.E.
Domestic Violence Hotlines	Buenos Aires (Argentina)	13.66***	−1.06	0.613***	−0.0513
	Colombia	66.03***	−3.089	0.821***	−0.0398
	Peru	151.7***	−20.08	0.15**	−0.0617
Emergency Lines	Costa Rica	−12.68***	−3.768	−0.103***	−0.0301
	Ecuador	−52.70***	−15.91	−0.175***	−0.0379
	Lima (Peru)	−100.3***	−7.693	−0.755***	−0.0489
Police Reports	Colombia	−67.52***	−5.861	−0.517***	−0.0491
	Ecuador	−18.72***	−4.055	−0.533***	−0.0677
	Uruguay	−15.12***	−4.713	−0.0825***	−0.0254

*Source:* Authors’ calculations based on data from the following sources: Colombia: Policía Nacional (police reports) and Línea 155 (domestic violence hotline); Uruguay: Ministerio del Interior (police reports); Ecuador: Fiscalía General (police reports) and ECU911 (emergency line); Costa Rica: Ministerio de Seguridad and Instituto Nacional de Mujeres (emergency line); Lima, Peru: Línea 105 (emergency line) and Línea 100 (domestic violence hotline); Buenos Aires, Argentina: Línea 137 (domestic violence hotline). *Note:* The figure presents the results of the OLS estimation of Eq. (1) for each of the datasets examined. We use daily data from January 1 to June 30 for the available years (see Section III for details) We estimate heteroscedasticity robust standard errors. Stars denote statistical significance: * 10 percent level. ** 5 percent level. *** 1 percent level.

We find significant increases in the number of calls to domestic violence hotlines in the three countries examined. In Buenos Aires, calls to the domestic violence hotline increased 84 percent; in Colombia, calls to Línea 155 rose 127 percent; and, in Peru, calls to Línea 100 increased 16 percent.^15^ In turn, we observe significant decreases in the number of incidents reported to emergency lines and through official police or judicial complaints. The number of domestic violence calls to emergency lines decreased by 16 percent in Ecuador, 10 percent in Costa Rica, and 53 percent in Lima, Peru. Police or judicial complaints fell 40 percent in Colombia, 41 percent in Ecuador, and 8 percent in Uruguay. All these estimates are significant at the 1 percent level.

The wide range of effects observed across countries and reporting channels is aligned with the variety of impacts observed in the literature. In a recent meta-analysis of the effect of COVID-19-related restrictions on reported incidents of domestic violence, Piquero et al. (2021) identify 18 empirical studies reporting effects on different measures of domestic violence ranging from −77 to 75 percent.

Our results include both relatively established and novel findings. On the one hand, the observed increases in domestic violence hotlines and decreases in police complaints align with findings in previous studies. In Mexico, Silverio-Murillo et al. (2020) find that calls to domestic violence hotlines increased 13 percent, while police reports decreased 22 percent in the first months of the pandemic. Using data from Chicago (United States), Bullinger et al. (2021) find that domestic violence calls increased 7.5 percent but reported domestic-related crimes fell by 8.7 percent after the introduction of stay-at-home orders.^16^ On the other hand, the fall in the number of calls to emergency lines has rarely been reported in other studies. For example, using data from different cities in the United States, Leslie and Wilson (2020), Mohler et al. (2020), Sanga and McCrary (2020) find increases ranging from 10 to 17 percent in police calls for service for domestic violence during the first weeks of the pandemic. A noteworthy exception is the work by Richards et al. (2021), who examine both police calls for service and calls to domestic violence hotlines in seven U.S. cities and find mostly increases in calls to domestic violence hotlines and mixed results for calls to the police.

These results prompt the question as to what characteristics of emergency lines and domestic violence hotlines could explain the divergent dynamics during the first months of the pandemic. The distinct path of the calls to emergency lines and calls to domestic violence hotlines might be related to the different response protocols of these two services. Generally, emergency lines are supposed to deal with the “most urgent” (even life-threatening) situations and domestic violence hotlines with less urgent cases, although, in practice, there might be overlap. The response protocols are designed in accordance with these objectives and, therefore, vary greatly across types of hotlines. The characteristics of the police and legal actions triggered by a call may affect the victim’s willingness to report (Goodmark, 2018). If a domestic violence hotline offers psychological support and provides the victim information without prompting legal actions or police proceedings, the perceived cost of reporting an incident is likely to be small. If, on the contrary, a call to an emergency line to report a domestic violence incident might translate into losing control of the legal process, victims might be deterred from reporting. In addition to this, victims’ perceptions of the costs (and benefits) of reporting depend on their trust in the police or the agency in charge of the hotline (Boateng, 2018). The relatively low levels of trust in the police in Latin American countries might exacerbate the perceived differences in the relative costs of using these alternative reporting channels. A recent opinion survey (Gallup, 2018) shows that only 42% of respondents in the region report trusting the local police, well below the levels observed in Western Europe (80%) and the United States and Canada (82%). In short, the less discreet response protocols applied by emergency lines and the low levels of trust in the police might explain why victims in the countries examined chose alternative channels to report domestic violence incidents during the pandemic.

## Shifting Patterns: How Did the Different Types of Reports Evolved During the Pandemic?

This section expands the previous analysis by separately examining different types of reports. First, we assess whether the pandemic’s impact on the number of domestic violence reports varied according to the type of violence reported. Second, we estimate the impact of the pandemic on domestic violence reports for different types of incidents based on the relationship between the victim and the perpetrator. Finally, we present evidence on the evolution of responses given to the users reporting domestic violence to Buenos Aires’ hotline.

### Type of Violence Reported

Some recent studies have shown that the pandemic induced greater increases in certain types of domestic violence than in others. Perez-Vincent et al. (2020) found a greater impact of the pandemic on psychological violence in Argentina, both in reports to the domestic violence hotline and in responses to an online victimization survey conducted in the first months of the pandemic. Arenas-Arroyo et al. (2020) also used an online victimization survey and found that the increase in domestic violence reports in Spain was explained solely by psychological violence. In this subsection, we analyze how the pandemic affected the number of domestic violence reports according to the type of violence reported, distinguishing between physical and psychological violence. To do so, we take advantage of the fact that records of calls to the domestic violence hotlines in Buenos Aires and Peru, calls to the emergency hotline in Costa Rica, and domestic violence legal complaints in Ecuador identify the type of violence reported in each recorded incident.^17^

**Fig. 7:**
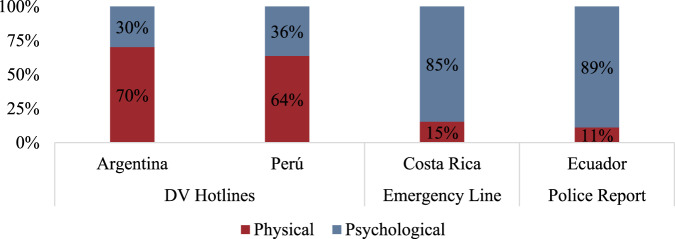
Type of Violence Reported before the Pandemic. *Source:* Author’s calculations based on data from Ecuador: Fiscalía General (police reports) and ECU911 (emergency line); Costa Rica: Ministerio de Seguridad and Instituto Nacional de Mujeres (emergency line); Peru: Línea 105 (emergency line); Buenos Aires, Argentina: Línea 137 (DV hotline)

Before the start of the pandemic, most calls to domestic violence hotlines reported physical violence incidents (70% in Argentina, 64% in Peru). Instead, most incidents recorded by emergency lines and police complaints were related to psychological violence (see Fig. 7 in the appendix). This composition changed drastically after the start of the pandemic.

Table 4 summarizes our main findings. In all cases, we find that reports of psychological violence led (or amplified) the changes observed in total reports. For the domestic violence hotline in Buenos Aires, we find a 41 percent increase in calls reporting incidents of physical violence and a 151 percent increase in calls related to psychological violence. Similarly, in the case of the domestic violence hotline in Peru, we observe an increase of 28 percent in calls related to psychological violence, while calls related to physical violence decreased by 18 percent.^18^ In turn, in the case of domestic violence calls to the emergency line in Costa Rica and legal complaints in Ecuador, where there was a drop in the number of reports, the drop was more pronounced for psychological violence. Reports of physical violence did not change in Costa Rica and fell 30 percent in Ecuador. In reports of psychological violence, both countries showed larger declines (−12 percent in 911 calls in Costa Rica, and −45 percent in legal complaints in Ecuador).

**Table 4:** The Impact of the pandemic on domestic violence reports, by type of violence

Source	Type of Violence	Estimated Effect (*β*)				
		Calls		IHS (Calls)		
		Coef.	S.E.	Coef.	S.E.	% Change
Buenos Aires (Domestic Violence Hotline)	Overall	13.66***	(1.060)	0.613***	(0.0513)	84
	Psychological	6.386***	(0.523)	0.924***	(0.0889)	151
	Physical	3.676***	(0.656)	0.344***	(0.0696)	41
Peru (Domestic Violence Hotline)	Overall	151.7***	(20.08)	0.150**	(0.0617)	16
	Psychological	56.20***	(6.507)	0.248***	(0.0648)	28
	Physical	−27.26***	(6.288)	−0.201***	(0.0640)	−18
Costa Rica (Emergency Line)	Overall	−12.68***	(3.768)	−0.103***	(0.0301)	−10
	Psychological	−11.85***	(3.157)	−0.124***	(0.0322)	−12
	Physical	0.308	(1.218)	0.0659	(0.0768)	6
Ecuador (Police Reports)	Overall	−18.72***	(4.055)	−0.533***	(0.0677)	−41
	Psychological	−17.35***	(3.193)	−0.599***	(0.0717)	−45
	Physical	−1.174	(0.998)	−0.344***	(0.107)	−30

*Source:* Authors’ calculations based on data from sources described in Section III

*Note:* The table presents the results of the OLS estimation of Eq. (1). We separately estimate the model for the different types of reports in each country, depending on the type of violence reported. We express the *β* coefficient as a percentage change following Bellemare and Wichman (2020). We use daily data from January 1 to June 30 for the available years (see Section III for more details). We estimate heteroscedasticity robust standard errors. Stars denote statistical significance: * 10 percent level. ** 5 percent level. *** 1 percent level

One possible explanation for the observed results is that the pandemic may have affected the relative incidence of different types of violence and the perceived costs and benefits of reporting them through different channels. These changes may have led to variations in the rate of reporting and the choice of reporting channels. Factors discussed in previous sections, such as fear of COVID-19 when entering a judicial process, mobility restrictions, or increased household economic insecurity may have reduced the likelihood a person would report a domestic violence incident to law enforcement authorities. It is possible that the impact of the pandemic on the decision to report or not (and the choice of the channel) also depended on the severity or type of incident. In the context of the pandemic, victims may have decided not to report incidents they considered less serious, especially if the decision to report may result in loss of control over the consequences or trigger prosecution.

The changes in reporting mechanisms documented in the previous subsection and the heterogeneity of the impact of the pandemic on the reporting of different types of violence provide insights into how the pandemic may have affected the incidence of different events, the willingness to report them, and the accessibility of different reporting channels.

Increases in calls to domestic violence hotlines suggest that these channels were the most appropriate for responding to the demand from domestic violence victims during the pandemic. In turn, the drop in calls to comprehensive emergency lines and legal complaints are consistent with an increase in the perceived (relative) cost of using these channels.

The changes in the composition of calls by type of violence could be due to victims considering these types of incidents less urgent and preferring to avoid initiating processes perceived as costly in turbulent and uncertain times such as those brought on by the pandemic. If so, the pandemic may have led domestic violence victims to gravitate toward domestic violence hotlines to the detriment of other channels, especially in those less urgent cases. This mechanism could explain the sharp decline in calls to general emergency hotlines and legal complaints about psychological violence and the increase in these calls to domestic violence hotlines.

### Type of relationship between victim and perpetrator

The changes in social dynamics brought about by the pandemic can be expected to have a different impact on domestic violence depending on the relationship between the victim and the perpetrator. Violence by close non-cohabitants, such as ex-partners, could be affected in a different way than violence by family members, partners, or other cohabitants.

On the one hand, the incidence of the most severe close non-cohabitant violence cases might increase. Early evidence on stalking victimization suggests that victims’ vulnerability was increased by the pandemic, as the lockdown made their whereabouts easier to monitor (Bracewell et al., 2020). On the other hand, mobility restrictions could decrease exposure to a particular kind of perpetrator. The pandemic could have played a mitigating role in ex-partner and other forms of non-cohabitant violence, which is in line with some early evidence (Ivandic et al., 2020).

This subsection focuses on the role that the type of relationship between victim and perpetrator played in shaping the impact of the pandemic on domestic violence reports. For this, we use data from Buenos Aires and Uruguay that provide this kind of information.^19^

We find that calls to Buenos Aires’ domestic violence hotline showed larger increases in reports of cohabitant violence (+78 percent) than reports of non-cohabitant violence (+42 percent), as reported in Table 5. This differential effect was also present in Uruguay, where only non-cohabitant domestic violence complaints (−17 percent) drove the observed overall decrease in domestic violence reports in the country (Table 5).

**Table 5:** The impact of the pandemic on domestic violence reports, cohabitant, and non-cohabitant violence

Source	Type of Perpetrator	Estimated Effect (*β*)				
		Calls		IHS(Calls)		
		Coef.	S.E.	Coef.	S.E.	% Change
Buenos Aires (Domestic Violence Hotline)	Overall	13.66***	(1.060)	0.613***	(0.0513)	84
	Cohabitant	7.328***	(0.726)	0.581***	(0.0633)	78
	Non-cohabitant	1.833***	(0.403)	0.351***	(0.0872)	42
Uruguay (Police Reports)	Overall	−15.12***	(4.713)	−0.0825***	(0.0254)	−8
	Cohabitant	−0.504	(3.581)	0.0069	(0.0341)	1
	Non-cohabitant	−13.37***	(2.797)	−0.18***	(0.0357)	−17

*Source:* Authors’ calculations based on data from the following sources: Uruguay: Ministerio del Interior (police reports); Ecuador: Fiscalía General (police reports) and ECU911 (emergency line); Buenos Aires, Argentina: Línea 137 (domestic violence hotline)

*Note:* The table presents the results of the OLS estimation of Eq. (1). In each country, we separately estimate the model for the different types of reports, depending on the relationship between victim and perpetrator. We express the λ*β* coefficient as a percentage change following Bellemare and Wichman (2020). We use daily data from January 1 to June 30 for the available years (see Section III for more details). We estimate heteroscedasticity robust standard errors. Stars denote statistical significance: * 10 percent level. ** 5 percent level. *** 1 percent level

The combined results of Argentina and Uruguay point to lower impacts—or even a mitigating role—of the pandemic in the incidence of non-cohabitant violence, in conformity with the mentioned findings by Ivandic et al. (2020). This behavior is also in line with exposure theory (Dugan et al. 1999) and recent findings from Perez-Vincent et al. (2020) that linked domestic violence increases with the rise in time couples spent together during the pandemic.

### Type of response

This subsection examines the evolution of calls depending on the response provided by the help service. We use the information included in the administrative records of calls to the domestic violence hotline in Buenos Aires (Line 137), which details the type of response given to each report of domestic violence. In particular, we distinguish between calls that resulted in providing information or orientation, and calls that ended up in a request for police intervention.^20^ Table 6 displays the impact of the first three months of the pandemic on the number of calls, differentiating by the type of action taken as a response to the call. The results show a sharp spike in calls that resulted in providing information or orientation (+147 percent). Though this increase account for the majority of the overall increase in calls, those that resulted in a request for police intervention also went up significantly (+59 percent). In line with the different dynamics observed across domestic violence hotlines, emergency lines, and police complaints, these results are consistent with the pandemic shifting demand for institutional help services toward providing information and away from more direct interventions.

**Table 6:** The impact of the pandemic on domestic violence reports, by response provided (línea 137, Buenos Aires, Argentina)

Type of Response	Estimated effect (*β*)				
	Calls		IHS (Calls)		
	Coef.	S.D.	Coef.	S.D.	% Change
All Calls	13.66***	−1.06	0.613***	−0.0513	84
Information Provided	9.242***	(0.701)	0.908***	(0.0802)	147
Police Intervention Requested	1.524***	−0.398	0.469***	−0.122	59

*Source:* Authors’ calculations based on data from the Línea 137 (domestic violence hotline) in Buenos Aires (Argentina).

*Note:* The table presents the results of the OLS estimation of Eq. (1). We estimate the model for the different types of calls. We express the *β* coefficient as a percentage change following Bellemare and Wichman (2020). We use daily data from January 1 to June 30 for 2017 to 2020. We estimate heteroscedasticity robust standard errors. Stars denote statistical significance: * 10 percent level. ** 5 percent level. *** 1 percent level.

## Conclusions

The stress, economic fluctuations, and mobility restrictions that followed the outbreak of the COVID-19 pandemic created conditions likely to increase domestic violence. This article examines how these circumstances changed the frequency and characteristics of domestic violence reports in a group of Latin American countries.

We find that the COVID-19 brought an increase in the demand for specialized domestic violence help services and a decrease in domestic violence reports through more traditional reporting channels, such as 911 calls and police or judicial complaints. The pandemic also changed the relative frequency of different types of reports (psychological vs. physical violence, and cohabitant vs. non-cohabitant violence).

As indicated in the Introduction, it is important to note our results refer to the pandemic’s impact on the *reporting* (and *recording*) of domestic violence, not necessarily on the *incidence* of domestic violence. Various factors could have affected the reporting and recording rates of these events without affecting their frequency (and vice versa). For example, a decline in the effective response rate of emergency services might lead to fewer events being recorded. Other factors could also have affected the ability and/or willingness of a victim to report domestic violence and could partly explain the observed shifts in the number of events registered by response services during the pandemic.

Our study contributes to the literature on the relationship between the pandemic and domestic violence in several relevant dimensions. First, we provide evidence on this relationship for multiple Latin American countries, broadening the geographic scope of the literature, which has focused primarily on the United States. Second, by using different sources of information, we provide a set of emerging insights into the heterogeneity of reporting dynamics during the pandemic. We find that the pandemic’s impact varied depending on the reporting channel, the type of violence reported, and the nature of the victim–perpetrator relationship. These heterogeneous effects help to make sense of previous ambiguous results in the literature. They also demonstrate domestic violence’s complexity and the need to continue to deepen the analysis to understand its drivers and the drivers of reporting.

The study also provides important implications for public policy. First, it highlights the need to provide a wide range of services to meet victims’ needs, which may vary according to contexts and circumstances. Domestic violence and its reporting are complex phenomena, which has only been made clearer by the pandemic. The supply of institutional help must be broad to cover different realities and be accessible to as many victims as possible. In this regard, the pandemic underscored the importance of specific hotlines for victims of domestic violence. In the first months of the pandemic, calls to domestic violence hotlines calls increased while reports through other channels fell. Although it is difficult to identify the specific reasons victims turned to hotlines, it seems clear that, as opposed to other services, hotlines managed to respond to victims’ demand for institutional help and bring it closer to them. This dynamic emphasizes the importance of developing a domestic violence hotline in those countries where one does not yet exist and strengthening existing ones.

Domestic violence is a pressing public policy issue in LAC. Governments and communities require a thorough understanding of this problem to address it effectively and efficiently. This work is one of the first attempts to identify, consolidate, and analyze sources of administrative information on domestic violence in several countries of the region. It is necessary to continue efforts to generate studies with new data sources and in more countries. Such efforts will help draw more solid conclusions about domestic violence dynamics and tailor institutional responses, both for the pandemic and beyond.

## Data Availability

We received most of the data used for the analysis carried out in the paper from government institutions, which provided it for this research. We do not have the explicit authorization to share them. We provide the names and references of the institutions that shared the data in the manuscript, and we can help researchers interested in accessing the data to contact these institutions and request the data. In the case of the publicly available data (Argentina and Peru’s domestic violence hotlines), we provide the link to the data in the paper.

## Appendix: Classification of Domestic Violence Reports

(Tables 7–11, Figs. 5–7)

**Table 7:** Calls to buenos aires domestic violence hotline línea 137: correspondence categories

Dimension	Categories in dataset	Authors’ categories
Type of Violence	Física	Physical
	Física y psicológica	
	Psicológica	Psychological
	No es un caso de violencia familiar	Not DV
	No aplica	
	Económica y/o Sexual (en ocasiones combinadas con violencia física y psicológica)	Other types
Type of Response	Comunicación con Equipos Móviles	Police intervention
	Desplazamiento de un Equipo Móvil a donde se encontraba la/s víctima/s	
	Está interviniendo o se deriva a otra institución	
	Intervención Equipos Móviles a donde se encontrara la/s víctima/s	
	Se planificó intervención para otro momento	
	Llamante solicitó información y/o orientación	Information provision
Relationship between Victim and Perpetrator	Esposo/a	Cohabitant
	Abuelo/a	
	Pareja	
	Hermano/a	
	Hijo/a	
	Madre	
	Padre	
	Padrastro	
	Pareja del padre /madre	
	Otro/a conviviente	
	Ex pareja	Non-cohabitant
	Ex esposo/a	
	Otro/a no conviviente

**Table 8:** Ecuador police reports: correspondence categories

Dimension	Categories in Dataset	Authors’ Categories
Type of Violence	Violencia psicológica contra la mujer o miembros del núcleo familiar	Psychological
	Violencia física contra la mujer o miembros del núcleo familiar	Physical

**Table 9:** Domestic violence calls to costa rica’s 911 emergency line: correspondence categories

Dimension	Categories in Dataset	Authors’ Categories
Type of Violence	Intrafamiliar niño/a psicológica	Psychological
	Intrafamiliar adulto/a mayor psicológica	
	Intrafamiliar hombre psicológica	
	Intrafamiliar mujer psicológica	
	Intrafamiliar niño/a física	Physical
	Intrafamiliar adulto/a mayor física	
	Intrafamiliar hombre física	
	Intrafamiliar mujer física

**Table 10:** Calls to peru’s domestic violence hotline, línea 100: correspondence categories

Dimension	Categories in Dataset	Authors’ Categories
Type of Violence	Psicológica	Psychological
	Violencia Psicológica	
	Física	Physical
	Violencia Física

**Table 11:** Uruguay police reports: correspondence categories

Dimension	Categories in Dataset	Authors’ Categories
Relationship between Victim and Perpetrator	Familiar	Cohabitant
	Pareja	
	Menor	
	Ex pareja	Non-cohabitant
	Otros	

## Footnotes

1 The exact date mobility restrictions were imposed varied across countries. We provide further detail in Section IV.

2 While these factors could have affected various victim response services, they not necessarily led to declines in their effective response rate. For example, Ecuador’s emergency line maintained a constant and high response rate throughout 2020 (around 98 percent). Information obtained from ECU911 (Ecuador) and the 911 emergency line in Costa Rica. Information obtained from ECU911 (Ecuador) and the 911 emergency line in Costa Rica.

3 For example, in Australia, there are indications that, although violence increased during the pandemic, victims were more reluctant to seek help for fear of COVID-19 (Payne & Morgan, 2020).

4 Perez-Vincent et al. (2020, chapter 1) examine the evolution of calls during the first month of the pandemic in the City of Buenos Aires, Argentina. The analysis for the City of Buenos Aires presented in our paper differs in two main ways: First, we extend the period examined until June 2020 (adding two more months into the pandemic). Second, we assess how the pandemic’s impact varied depending on the type of relationship between the victim and the perpetrator (cohabitant vs. non-cohabitant). Agüero (2021) examined the evolution of calls to the domestic violence helpline in Perú (Línea 100). Our study uses daily data (as opposed to monthly data used by Agüero, 2021) for a longer timeframe, which allows us to estimate the short-term impact of the pandemic more precisely. We also examine heterogeneities not examined by the previous paper and complement the analysis with data from the national emergency line (Línea 105).

5 Effective calls are those in which the caller and the operator communicate and exchange information. Most databases include information only on effective calls and exclude calls that are not answered or those in which the caller does not speak or does not provide any information.

6 In most Latin American countries, administrative records on domestic violence incidents are not publicly available. We contacted different institutions that register domestic violence incidents (mainly, emergency services, law enforcement agencies, and women’s ministries), requesting access to the data. We thank the institutions that generously shared their data for the purpose of this research.

7 We obtained this dataset from the official data repository of the Ministry of Justice and Human Rights: http://datos.jus.gob.ar/dataset/linea-137-victimas-de-violencia-familiar (accessed March 30, 2021).

8 Available at https://portalestadistico.aurora.gob.pe/bases-de-datos-2018/ for 2018, https://portalestadistico.aurora.gob.pe/bases-de-datos-2019/ for 2019 and https://portalestadistico.aurora.gob.pe/bases-de-datos-2020/ for 2020 (Accessed on May, 2021).

9 Available at https://www.google.com/covid19/mobility (accessed on January 15, 2021). The Google Mobility Report tracks how people’s time spent at locations associated with retail and recreation, grocery and pharmacy, parks, transit stations, workplaces and residences changed in 2020 compared with the average for each day of the week that was observed between January 3, 2020 and February 6, 2020. This study looks at the residential percent change or time spent at home in our terms.

10 Available at: https://github.com/OxCGRT/covid-policy-tracker/raw/master/data/timeseries/OxCGRT_timeseries_all.xlsx (accessed on January 15, 2021). OxCGRT collects publicly available information on 20 indicators of government responses. The methodology for the construction of the indexes is available here: https://github.com/OxCGRT/covid-policy-tracker/blob/master/documentation/index_methodology.md (accessed on December 29, 2021).

11 https://ourworldindata.org/coronavirus/country/costa-rica?country=~CRI.

12 Following the discussion in the previous section, we consider these dates as the start of the first lockdown in 2020 in each country: Ecuador: March 12; Peru, Costa Rica and Uruguay: March 16; Buenos Aires (Argentina): March 20; Colombia: March 25.

13 To interpret *β* as a percentage change, we followed Halvorsen and Palmquist (1980) and used the correction suggested by Kennedy (1981) as explained in Bellemare and Wichman (2020): % *Change* = *Exp*(*β*−0.5(*Var*(*β*)))−1. We present the *β* estimate and the percent change in the tables.

14 For this process, we needed to choose a number of lags beyond which autocorrelation could be ignored. There is not one single way to define the correct number of periods beyond which autocorrelation is negligible. As pointed out by Greene (2000), most studies use *Sample Length*^1/4^ as the limit. For robustness, we evaluated the results using 1 to 7 lags, which always included the mentioned *Sample Length*^1/4^.

15 Perez-Vincent et al. (2020, chapter 1) examine the evolution of calls during the first month of the pandemic in the City of Buenos Aires, Argentina. They find a 28 percent increase in the number of calls in that period. We find a larger increase in the number of calls, which confirm that the increase in calls became more pronounced after the first month. Agüero (2021) examined the evolution of calls to the domestic violence helpline in Perú (Línea 100) using monthly data. He finds that calls increased 48 percent between April and July 2020. We use a more recent database that provides daily data and allows us to identify that start of the pandemic and estimate the subsequent change in the number of calls more precisely. We find an average increase of 16% for the period March 16-June 30, which captures an initial fall during the first weeks of the pandemic (as shown in Fig. 3).

16 Additionally, in the United States, the total number of contacts to the National Domestic Violence Hotline increased 9 percent in the first two months of the pandemic: https://www.thehotline.org/resources/a-snapshot-of-domestic-violence-during-covid-19/ (accessed December 20, 2021).

17 See Tables 7–11 in the appendix for classification details.

18 For Peru’s domestic violence hotline, calls that did not identify type of violence (registered as *otra consulta*, or other query) represent a large share of the total calls and grew from approximately a quarter of the records to almost half of all calls during the pandemic, leading the overall increase in total calls.

19 See Tables 7–11 and 11 in the appendix for classification details.

20 See Tables 7 in the appendix for classification details.

## Code availability

We are happy to share the replication programs and a detailed description of the data upon request.
